# Publisher Correction: Targeting GRPR for sex hormone-dependent cancer after loss of E-cadherin

**DOI:** 10.1038/s41586-025-09353-9

**Published:** 2025-07-14

**Authors:** Jérémy H. Raymond, Zackie Aktary, Marie Pouteaux, Valérie Petit, Flavie Luciani, Maria Wehbe, Patrick Gizzi, Claire Bourban, Didier Decaudin, Fariba Nemati, Igor Martianov, Irwin Davidson, Catherine-Laure Tomasetto, Richard M. White, Florence Mahuteau-Betzer, Béatrice Vergier, Lionel Larue, Véronique Delmas

**Affiliations:** 1 Normal and Pathological Development of Melanocytes, Institut Curie, PSL Research University, INSERM U1021, Orsay, France; 2 Université Paris-Saclay, CNRS UMR 3347, Orsay, France; 3 CNRS UMS3286, Plateforme de Chimie Biologique Intégrative de Strasbourg, Strasbourg University/Strasbourg Drug Discovery and Development Institute (IMS), Strasbourg, France; 4 Laboratory of Preclinical Investigation, Department of Translational Research, Institut Curie, PSL University Paris, Paris, France; 5 IGBMC, CNRS UMR7104, INSERM U1258, Université de Strasbourg, Illkirch, France; 6 Ludwig Institute for Cancer Research, University of Oxford, Oxford, UK; 7 CNRS UMR9187, INSERM U1196, Chemistry and Modeling for the Biology of Cancer, Institut Curie, Université PSL, Orsay, France; 8 Université Paris-Saclay, CNRS UMR 9187, INSERM U1196, Orsay, France; 9 Service de pathologie CHU de Bordeaux et Eq. Translational Research on Oncodermatology and Orphean Skin Diseases (TRIO2) BoRdeaux Institute of onCology (BRIC) UMR 1312, INSERM/Université de Bordeaux, Bordeaux, France

**Keywords:** Melanoma, Transcription

Correction to: *Nature* 10.1038/s41586-025-09111-x Published online 11 June 2025

This article was originally published under standard Springer Nature license (© The Author(s), under exclusive licence to Springer Nature Limited). It is now available as an open-access paper under a Creative Commons Attribution 4.0 International license, © The Author(s).

