# Supplementary material for: Targeting GRPR for sex hormone-dependent cancer after loss of E-cadherin
Source: Nature. 2025 Jun 11;643(8072):801–9. doi: 10.1038/s41586-025-09111-x (PMC12267067; doi:10.1038/s41586-025-09111-x)
Supplement: Supplementary file 1 — Original source images for all data obtained by electrophoretic separation. Data are organized b figure, with all replicates included. The data displayed in each Figure and Extended Figure are indicated with red boxes. [file 41586_2025_9111_MOESM1_ESM.pdf]

nature portfolio

https://doi.org/10.1038/s41586-025-09111-x

Supplementary information

Targeting GRPR for sex hormone-dependent cancer after loss of E-cadherin

In the format provided by the authors and unedited

Blots used in figures are indicated with a red square

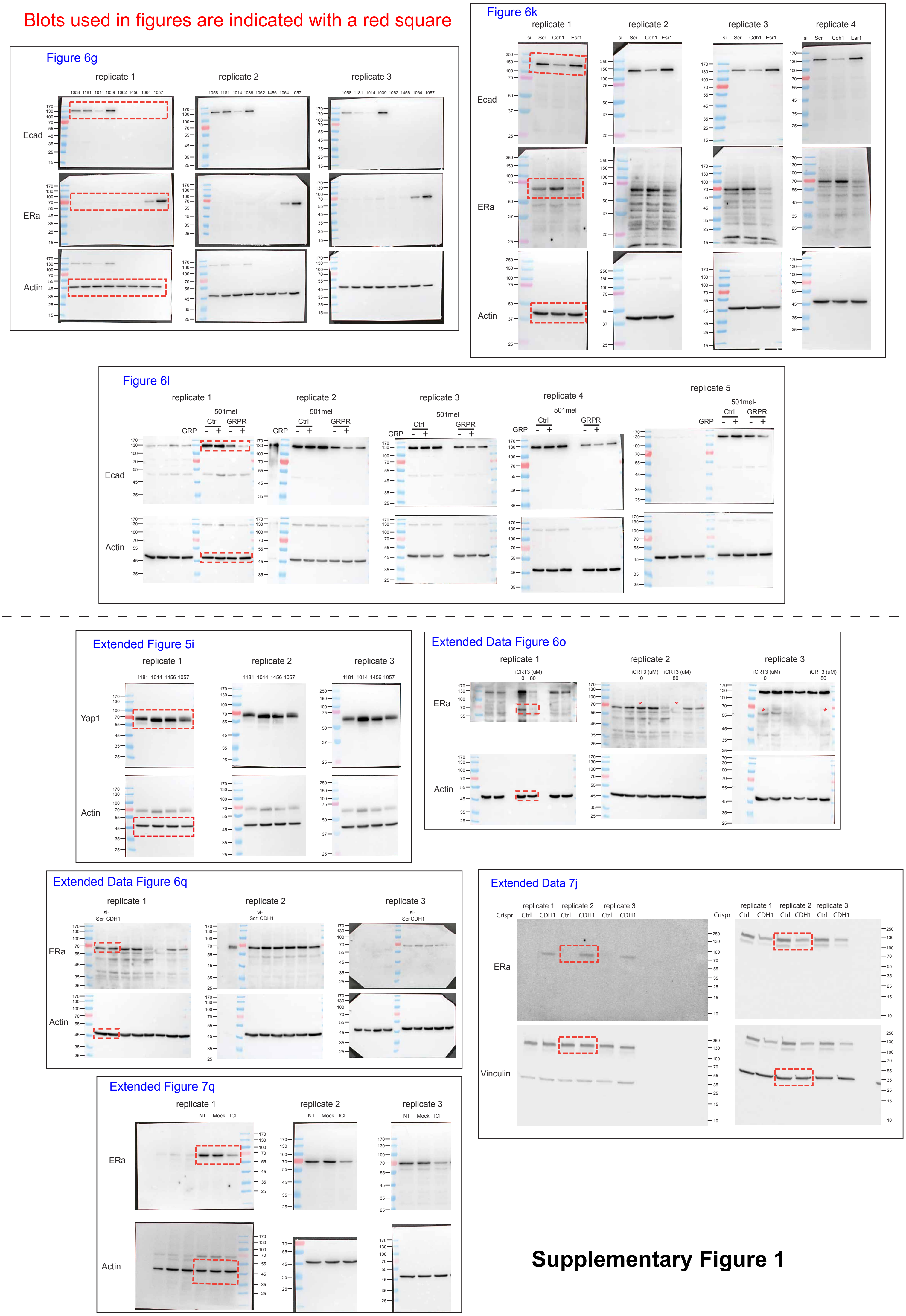

Supplementary Figure 1
