# Supplementary material for: Targeting GRPR for sex hormone-dependent cancer after loss of E-cadherin
Source: Nature. 2025 Jun 11;643(8072):801–9. doi: 10.1038/s41586-025-09111-x (PMC12267067; doi:10.1038/s41586-025-09111-x)
Supplement: Supplementary file 2 — Reporting Summary [file 41586_2025_9111_MOESM2_ESM.pdf]

nature portfolio

Corresponding author(s): Lionel LARUE

Last updated by author(s): 22/04/2025

# Reporting Summary

Nature Portfolio wishes to improve the reproducibility of the work that we publish. This form provides structure for consistency and transparency in reporting. For further information on Nature Portfolio policies, see our Editorial Policies and the Editorial Policy Checklist.

## Statistics

For all statistical analyses, confirm that the following items are present in the figure legend, table legend, main text, or Methods section.

| n/a | Confirmed | |
|---|---|---|
| ☐ | ☒ | The exact sample size (*n*) for each experimental group/condition, given as a discrete number and unit of measurement |
| ☐ | ☒ | A statement on whether measurements were taken from distinct samples or whether the same sample was measured repeatedly |
| ☐ | ☒ | The statistical test(s) used AND whether they are one- or two-sided<br>*Only common tests should be described solely by name; describe more complex techniques in the Methods section.* |
| ☐ | ☒ | A description of all covariates tested |
| ☐ | ☒ | A description of any assumptions or corrections, such as tests of normality and adjustment for multiple comparisons |
| ☐ | ☒ | A full description of the statistical parameters including central tendency (e.g. means) or other basic estimates (e.g. regression coefficient) AND variation (e.g. standard deviation) or associated estimates of uncertainty (e.g. confidence intervals) |
| ☐ | ☒ | For null hypothesis testing, the test statistic (e.g. *F*, *t*, *r*) with confidence intervals, effect sizes, degrees of freedom and *P* value noted<br>*Give P values as exact values whenever suitable.* |
| ☒ | ☐ | For Bayesian analysis, information on the choice of priors and Markov chain Monte Carlo settings |
| ☐ | ☒ | For hierarchical and complex designs, identification of the appropriate level for tests and full reporting of outcomes |
| ☐ | ☒ | Estimates of effect sizes (e.g. Cohen's *d*, Pearson's *r*), indicating how they were calculated |

*Our web collection on statistics for biologists contains articles on many of the points above.*

## Software and code

Policy information about availability of computer code

| | |
|---|---|
| Data collection | Epidemiological data were dowloaded from the Global Cancer Observatory repository on the International Agency for Research on Cancer (https://gco.iarc.fr). Transcriptomic and genomic data were collected using the open source R package TCGAbiolinks (v 2.28.3) or by directly downloading them from cBioportal (http://www.cbioportal.org/). Kinase activity were acquired using the PamStation PS12 (PamGene International B.V.). |
| Data analysis | No custom codes were developed to analyse the data associated with this manuscript. All codes used originate from well-established R packages: DEseq2 (v1.40.2), edgeR (v3.42.4), sva (v3.48.0), vsn (v3.68.0), ChIPseeker (v1.36.0). Functions from these packages were used using default parameters except if stated otherwise in the Material and Methods section of this manuscript. Data were aligned using RNA-STAR (2.7.11) Bowtie (2.5.3) or BWA (0.7.19). MarkDuplicates (v3.1.1.0) was used to remove PCR duplicates. Peaks were called with MACS2 (v2.2.7.1). TCGA data were downloaded using TCGAbiolinks (v2.28.3).<br>GSEA analyses were performed using the GSEA software from the Broad Institute (v4.0.3). PamGene Kinase assays were analysed using the Bionavigator software, PamGene International B.V.). Automatic counting of invading cells was done using an macro (https://zenodo.org/records/14509394) on ImageJ/Fiji (v2.14.0/1.54f). Data were represented using GraphPad Prism 10 (v10.2.3) or R (4.4.0) and the package ggplot2 (v3.5.1). |

For manuscripts utilizing custom algorithms or software that are central to the research but not yet described in published literature, software must be made available to editors and reviewers. We strongly encourage code deposition in a community repository (e.g. GitHub). See the Nature Portfolio guidelines for submitting code & software for further information.

## Data

Policy information about availability of data

All manuscripts must include a data availability statement. This statement should provide the following information, where applicable:

- Accession codes, unique identifiers, or web links for publicly available datasets
- A description of any restrictions on data availability
- For clinical datasets or third party data, please ensure that the statement adheres to our policy

All sequencing data generated with this manuscript were deposited on the relevant platform. RNA-seq and ChIP-seq data were deposited on GEO at the National Center for Biotechnology Information under the SuperSeries GSE218588 (https://www.ncbi.nlm.nih.gov/geo/query/acc.cgi?acc=GSE218588). That includes: the mouse tumour RNA-seq under accession number GSE218532 (https://www.ncbi.nlm.nih.gov/geo/query/acc.cgi?acc=GSE218532), the mouse cell line RNA-seq under accession number GSE218586 (https://www.ncbi.nlm.nih.gov/geo/query/acc.cgi?acc=GSE218586) and the human cell line data are deposited under accession number GSE218530 (https://www.ncbi.nlm.nih.gov/geo/query/acc.cgi?acc=GSE218530). The ChIP-seq data are available under accession number GSE237500 (https://www.ncbi.nlm.nih.gov/geo/query/acc.cgi?acc=GSE237500). Whole exome sequencing of the mouse melanoma cell lines are available from SRA under the bioproject PRJNA904253 (https://dataview.ncbi.nlm.nih.gov/object/40767225) Kinase assay raw data were deposited on Mendeley at the following DOI 10.17632/nwkpyr2nmh.1 (Currently under publication, will provide the final DOI shortly). All other data supporting the findings of this study are available on reasonable request. Source data are provided with this paper.
The following datasets from the literature were used: ChIP-seq of ERa in mouse (GSM894054, https://www.ncbi.nlm.nih.gov/geo/query/acc.cgi?acc=GSM894054) and human (GSM798434, https://www.ncbi.nlm.nih.gov/geo/query/acc.cgi?acc=GSM798434), of b-catenin in mouse (GSM980186, https://www.ncbi.nlm.nih.gov/geo/query/acc.cgi?acc=GSM980186) and human (GSM1579346, https://www.ncbi.nlm.nih.gov/geo/query/acc.cgi?acc=GSM1579346) and of H3K27Ac in human MCF7 (GSM2175784, https://www.ncbi.nlm.nih.gov/geo/query/acc.cgi?acc=GSM2175784). ATAC-seq data from the MCF7 cell line originate from GSM2645717 (https://www.ncbi.nlm.nih.gov/geo/query/acc.cgi?acc=GSM2645717). The 3D chromatin interactions were downloaded from GSE207828 (https://www.ncbi.nlm.nih.gov/geo/query/acc.cgi?acc=GSE207828) for mouse and from ENCODE accession number ENCFF804SET (https://www.encodeproject.org/experiments/ENCSR549MGQ/) and from GSE52457 (https://www.ncbi.nlm.nih.gov/geo/query/acc.cgi?acc=GSE52457) for human. Expression data from acral melanoma is from GSE190113 (https://www.ncbi.nlm.nih.gov/geo/query/acc.cgi?acc=GSE190113). TCGA datasets were accessed through TCGABiolinks or CBioPortal (https://www.cbioportal.org/).

All data have been aligned to the human reference genome GRCh38 (hg38 gencode 42 version GRCh38.p13) and the mouse reference genome mm10 gencode 13 version GRCm38.p5.

## Research involving human participants, their data, or biological material

Policy information about studies with human participants or human data. See also policy information about sex, gender (identity/presentation), and sexual orientation and race, ethnicity and racism.

| | |
|---|---|
| Reporting on sex and gender | Patient gender was defined by their self reporting. |
| Reporting on race, ethnicity, or other socially relevant groupings | No information has been collected on race, ethnic criteria or any socially relevant grouping because the collection of this type of data is legally prohibited in France (Law no. 78-17 of 6 January 1978 on data processing, data files and individual liberties, Article 226-19 of the Criminal Code) |
| Population characteristics | The population had an average median of 58 years old (25% interquartile range: 48-66). Their genotypes (52% BRAF mutated, 17% NRAS mutated) represent the known frequency of mutation this two main driver genes. |
| Recruitment | The recruitment was made retrospectively on the available sample from the tissue collection of the Rennes and Bordeaux Hospitals. |
| Ethics oversight | The retrospective study on lung human melanoma metastases was approved by the ethics committee. The non-opposition or consent (before or after 2004, respectively) of patients for the use of their biological material and data was obtained according to the bioethics law of 2004. This study was approved by the C.H.U. de Bordeaux. |

Note that full information on the approval of the study protocol must also be provided in the manuscript.

# Field-specific reporting

Please select the one below that is the best fit for your research. If you are not sure, read the appropriate sections before making your selection.

☒ Life sciences ☐ Behavioural & social sciences ☐ Ecological, evolutionary & environmental sciences

For a reference copy of the document with all sections, see nature.com/documents/nr-reporting-summary-flat.pdf

# Life sciences study design

All studies must disclose on these points even when the disclosure is negative.

| | |
|---|---|
| Sample size | The required number of mouse per group was determined by calculating the minimum number of subject to have a sufficient study power. The statistical parameter applied were: |

- We expect a minimum of 30% variation in between our conditions
- The type 1 error set at 5%
- The power was set at 80%.

Based on this number it was calculated that we needed 5 mice per group.

| | |
|---|---|
| Data exclusions | No data were excluded during this work |
| Replication | All in vitro experiments were successfully performed at least with three independent biological replicates. For the experiments involving cell lines, each replicate corresponds to different passages. |
| Randomization | For every in vivo experiment, mouse were blind randomized using an online randomization software (https://www.randomizer.org/). |
| Blinding | Experiments were not performed in a blind manner. However, biological replicates were performed by different individual without prior knowledge of the results of the others. |

# Reporting for specific materials, systems and methods

We require information from authors about some types of materials, experimental systems and methods used in many studies. Here, indicate whether each material, system or method listed is relevant to your study. If you are not sure if a list item applies to your research, read the appropriate section before selecting a response.

## Materials & experimental systems

| n/a | Involved in the study | |
|---|---|---|
| ☐ | ☒ | Antibodies |
| ☐ | ☒ | Eukaryotic cell lines |
| ☒ | ☐ | Palaeontology and archaeology |
| ☐ | ☒ | Animals and other organisms |
| ☐ | ☒ | Clinical data |
| ☒ | ☐ | Dual use research of concern |
| ☒ | ☐ | Plants |

## Methods

| n/a | Involved in the study | |
|---|---|---|
| ☐ | ☒ | ChIP-seq |
| ☒ | ☐ | Flow cytometry |
| ☒ | ☐ | MRI-based neuroimaging |

## Antibodies

Antibodies used

o YAP: clone D8H1X, Cell Signaling Technology, Cat#14074, RRID: AB_2650491
o Gastrin-Releasing Peptide Receptor (GRPR), Acris Antibodies, Cat#SP4337P, RRID: AB_1001744
o E-cadherin, Leica Biosystems Cat#E-CAD-L-CE
o E-cadherin, BD transduction laboratories, Cat#610182, RRID: AB_397581
o Histone H3K27ac, Active Motif, Cat#39133, RRID: AB_2722569
o Vinculin, Cell Signaling Technology, Cat#4650, RRID: AB_10559207
o ERa,Invitrogen, Cat#MA1-27107, RRID: AB_780508
o B-actin, Sigma-Aldrich, Cat#A5441, RRID: AB_476744
o goat anti-rabbit Alexa fluor 594, Invitrogen, Cat#A-11012, RRID: AB_2534079

Validation

Validation of the antibodies used for this study:
o YAP: this antibody has been validated by the knock-out of YAP1 in Hela cells which leads to the loss of detection of the protein in cell whereas YAP remained detectable in the control cells.
o GRPR: we validate this antibody human tissues by incubating tissues known to express or not GRPR at the RNA level with the antibody. Only the tissues expressing GRPR were stained in immunohistochemistry.
o E-cadherin: we validate this antibody human tissues by incubating tissues known to express or not Ecad at the RNA level with the antibody. Only the tissues expressing Ecad were stained in immunohistochemistry.
o Histone H3K27ac: This antibody was validated by the manufacturer notably to for its use in ChIP-seq.
o anti-B-actin was validated by the manufacturer for their usage in western blot (“The antibody specifically labels β-actin in a wide variety of tissues and species using immunoblotting (42 kDa)”, and “species reactivity: sheep, carp, feline, chicken, rat, mouse, Hirudo medicinalis, rabbit, canine, pig, human, bovine, guinea pig”). This antibody is also highly used in the litterature (>890 citations)
o Vinculin antibodies were validated by the manufacturer for their usage in western blot. Both of those antibodies are widely used in the literature in this application (>390 citations).
o ERa the antibody was validated by the manufacturer “Estrogen Receptor alpha Antibody (MA1-27107) Antibody specificity was demonstrated by detection of differential basal expression of the target across cell lines and tissues owing to their inherent genetic constitution”. We further validated the antibody by immunobloting ERa in our mouse melanoma cell line and showed that the protein expression correlates with the mRNA expression (Figure 5d-e). The specificity was proven by knocking-down Esr1 expression and showing that it decreases ERa signal detected by immunoblotting (Figure 5i).

## Eukaryotic cell lines

Policy information about cell lines and Sex and Gender in Research

Cell line source(s) — Mouse cell lines were established by the laboratory during this study.

| | |
|---|---|
| Cell line source(s) | Human melanoma cell lines were described previously by the laboratory (Rambow et all. Cell Report, 2015). MCF7 were gifted by the Dutreix laboratory (Institut Curie, Orsay, France). |
| Authentication | Cells were authenticated by the STR profiling method. |
| Mycoplasma contamination | All cells were frequently tested for mycoplasma contamination. None of them were contaminated. |
| Commonly misidentified lines (See ICLAC register) | No commonly misidentified cell lines were used in this study. |

## Animals and other research organisms

Policy information about studies involving animals; ARRIVE guidelines recommended for reporting animal research, and Sex and Gender in Research

| | |
|---|---|
| Laboratory animals | This study was conducted using mouse of the following strains:<br>- C57BL/6J, RRID:IMSR_JAX:000664, Charles River, aged of 8 weeks when included in the protocol<br>- NSG (NOD.Cg-Prkdcscid Il2rgtm1Wjl/SzJ), RRID:IMSR_JAX:005557, Charles River, aged of 8 weeks when included in the protocol<br><br>As well as te following transgenic models:<br>-Tyr::CreA (B6.Cg-Tg(Tyr-cre)1Lru/J), Larue Lab, RRID: IMSR_JAX:029788Info<br>- Tyr::CreB (Delmas et al., Genesis. (2003), 36, 73-80. PMID: 12820167)<br>-Tyr::NRAS (Tg(Tyr-NRAS*Q61K)1Bee), Beerman Lab, RRID:MGI:5515808<br>-Cdkn2a+/- (Cdkn2atm1Rdp) De Pinho Lab, RRID:IMSR_NCIMR:01XB1<br>-Cdh1F/+ B6.129-CDH1tm2kem/J), Kemler Lab, RRID:IMSR_JAX:005319<br>All transgenic models were included in the study from their birth date up to 120 weeks if no melanoma appeared (see Figure 1F and associated source data for per mouse details). All these transgenic mouse lines have been backcrossed at least 10 times with C57BL/6J mouse (including male and female mice).<br>Mice were housed in a spf certified animal facility with a 12-hour light/dark cycle in a temperature-and humidity-controlled room (22 ±1°C and 60% respectively) with free access to water and food. |
| Wild animals | No wild animals were involved in this study. |
| Reporting on sex | For the transgenic animals, all sexes were considered. For the WT animals used for cell injections, the sex of the animal correspond to the sex of the injected cell line. |
| Field-collected samples | No samples were collected from the field. |
| Ethics oversight | Animal care, use, and all experimental procedures were conducted in accordance with recommendations of the European Community (86/609/EEC) and Union (2010/63/UE) and the French National Committee (87/848). Animal care and use were approved by the ethics committee of the Curie Institute in compliance with institutional guidelines. Experimental procedures were carried out under the approval of the ethics committee of the Institut Curie CEEA-IC #118 (CEEA-IC 2016-836 001) in compliance with international guidelines |

Note that full information on the approval of the study protocol must also be provided in the manuscript.

## Clinical data

Policy information about clinical studies

All manuscripts should comply with the ICMJE guidelines for publication of clinical research and a completed CONSORT checklist must be included with all submissions.

| | |
|---|---|
| Clinical trial registration | N/A |
| Study protocol | N/A |
| Data collection | N/A |
| Outcomes | N/A |

## Plants

| | |
|---|---|
| Seed stocks | N/A |
| Novel plant genotypes | N/A |
| Authentication | N/A |

# ChIP-seq

## Data deposition

☒ Confirm that both raw and final processed data have been deposited in a public database such as GEO.

☒ Confirm that you have deposited or provided access to graph files (e.g. BED files) for the called peaks.

| | |
|---|---|
| Data access links<br>*May remain private before publication.* | https://www.ncbi.nlm.nih.gov/geo/query/acc.cgi?acc=GSE237500<br>token: mxuzyaekdvifnkz |
| Files in database submission | GSM7622286_1007_AC.bigwig<br>GSM7622287_1181_AC.bigwig<br>GSM7622288_1014_AC.bigwig<br>GSM7622289_1039_AC.bigwig<br>GSM7622290_1062_AC.bigwig<br>GSM7622291_1456_AC.bigwig<br>GSM7622292_1057_AC.bigwig<br>GSM7622293_1064_AC.bigwig<br>GSM7622294_1069_AC.bigwig<br>GSM7622295_Ecad_male_Input.bigwig<br>GSM7622296_Ecad_female_Input.bigwig<br>GSM7622297_dEcad_male_Input.bigwig<br>GSM7622298_dEcad_female_Input.bigwig<br>peaks_1007_H3K27ac.bed<br>peaks_1181_H3K27ac.bed<br>peaks_1014_H3K27ac.bed<br>peaks_1039_H3K27ac.bed<br>peaks_1062_H3K27ac.bed<br>peaks_1456_H3K27ac.bed<br>peaks_1057_H3K27ac.bed<br>peaks_1064_H3K27ac.bed<br>peaks_1069_H3K27ac.bed |
| Genome browser session<br>(e.g. UCSC) | http://genome.ucsc.edu/cgi-bin/hgTracks?<br>db=mm10&lastVirtModeType=default&lastVirtModeExtraState=&virtModeType=default&virtMode=0&nonVirtPosition=&po<br>sition=chrX%3A163191359%2D164097582&hgsid=1725092268_Ac6A1PXaGTMqNJPmaWuMKZOlYsEy |

## Methodology

| | |
|---|---|
| Replicates | We performed the ChIP-seq on two cell lines of each Cdh1 genetic status and of each sex. |
| Sequencing depth | AC-1062: 80186942 reads, 78% uniquely mapped<br>AC-1057: 85720740 reads, 80% uniquely mapped<br>AC-1064: 60195608 reads, 76% uniquely mapped<br>AC-1069: 97118449 reads, 72% uniquely mapped<br>AC-1007: 91033436 reads, 81% uniquely mapped<br>AC-1181: 55183731 reads, 72% uniquely mapped<br>AC-1456: 71277500 reads, 73% uniquely mapped<br>AC-1014: 83138809 reads, 74% uniquely mapped<br>AC-1039: 99758567 reads, 81% uniquely mapped<br>Input-1062: 21131673 reads, 58% uniquely mapped<br>Input-1181: 83424648 reads, 64% uniquely mapped<br>Input-1069: 24846923 reads, 39% uniquely mapped<br>Input-1014: 29629266 reads, 60% uniquely mapped<br>All reads were pair-ends, read length is 100bp |
| Antibodies | Histone H3K27ac antibody, Active Motif, Cat#39133, RRID: AB_2722569 |
| Peak calling parameters | Peak calling was performed using MACS2 (v2.2.7.1). Default parameters were used except for the following:<br>- Input files as paired-end BAM<br>- Effective genome size: 1.87e9 (M.musculus genome size)<br>- q-value threshold= 1e-05<br>- Band width for picking regions to compute fragment size: 300bp |
| Data quality | ChIP seq quality was assessed by the simple exponential smoothing method. Each ChIP fingerprint was plotted and it was verified that most of the reads correspond to a small part of the genome except for the input samples where reads were homogeneously distributed across the genome. For all samples, 50% of the reads were located in only 5% of the genome, which is sign of a strong and localized enrichments. For the input, these 50% of the reads are aligned to 20 to 35% of the genome. |
| Software | ChIP-seq were analysed using Galaxy. Fastq were trimmed using FASTQ trimmer and then align on the mm10 mouse genome using bowtie2. Peaks were called using MACS2 and the parameter defined above. For representation purpose, bigwig were generated. |

These bigwig were generated using the BamCoverage function from DeepTools. Bin size was set at 20 bases. Reads were normalized to bins per million and the scaling factor was set to 1.
